# The polarization modulation and fabrication method of two dimensional silica photonic crystals based on UV nanoimprint lithography and hot imprint

**DOI:** 10.1038/srep34495

**Published:** 2016-10-04

**Authors:** Shuai Guo, Chunhui Niu, Liang Liang, Ke Chai, Yaqing Jia, Fangyin Zhao, Ya Li, Bingsuo Zou, Ruibin Liu

**Affiliations:** 1 Beijing Key Laboratory of Nanophotonics and Ultrafine Optoelectronic Systems, Institute of Physics, Beijing Institute of Technology, Beijing 100081, China; 2 School of Instrument Science and Opto-electronic Engineering, Beijing University of Information Science & Technology, Beijing 100192, China; 3 Beijing institute of metrology, Beijing 100029, China

## Abstract

Based on a silica sol-gel technique, highly-structurally ordered silica photonic structures were fabricated by UV lithography and hot manual nanoimprint efforts, which makes large-scale fabrication of silica photonic crystals easy and results in low-cost. These photonic structures show perfect periodicity, smooth and flat surfaces and consistent aspect ratios, which are checked by scanning electron microscopy (SEM) and atomic force microscopy (AFM). In addition, glass substrates with imprinted photonic nanostructures show good diffraction performance in both transmission and reflection mode. Furthermore, the reflection efficiency can be enhanced by 5 nm Au nanoparticle coating, which does not affect the original imprint structure. Also the refractive index and dielectric constant of the imprinted silica is close to that of the dielectric layer in nanodevices. In addition, the polarization characteristics of the reflected light can be modulated by stripe nanostructures through changing the incident light angle. The experimental findings match with theoretical results, making silica photonic nanostructures functional integration layers in many optical or optoelectronic devices, such as LED and microlasers to enhance the optical performance and modulate polarization properties in an economical and large-scale way.

The silica (SiO_2_) layer is a basic dielectric layer in many semiconductor devices due to high-resistivity and dielectric strength to be used to confine electrons in field effect transistors (FET), light emitting diodes (LED) and metal-oxide-semiconductor field-effect transistors (MOSFET). Recently, due to excellent and special optical characteristics of silica photonic crystals, these are gradually used in optical data transmission, laser spectroscopy and tailoring light speed^1^,^2^,^3^,^4^,^5^. Ideally, silica crystal layers can act as both dielectric isolation and specific functional layers to modulate light in optoelectronic nano devices. Examples include antireflection coatings in solar cells^6^ and structured layers in vertical cavity surface emitting lasers (VCSELs)^7^. Generally, producing high-quality silica photonic structures is an expensive and time-consuming process for traditional crafts, including participation of Plasma-Enhanced Chemical Vapor Deposition (PECVD), Electron-Beam Lithography (EBL), Focused-Ion-Beam (FIB) and UV photolithography combined with etching processes^8^,^9^,^10^,^11^. The quality of 2-D silica crystals is subjective to controllability of details of each fabrication process, especially etching.

Nano imprint lithography (NIL) was proposed as one of the best nano-patterning techniques due to significant advantages: fast, low-cost, high-resolution, superior quality, high throughput, and large-scale patterning^12^,^13^,^14^,^15^,^16^. It has been applied in the manufacturing process of micro and nano-devices, such as photoelectric devices (solar cells^17^,^18^, light emitting diodes^19^,^20^), biology fields (biochips^21^) and optical components (polarizers^22^,^23^, photonic crystals^24^,^25^). However, harsh working conditions and low yields make it difficult to apply NIL in many application fields. In this paper, a Substrate Conformal Imprint Lithography (SCIL) technique^26^ and silica nanoimprint resist is utilized to fabricate highly-ordered silica photonic crystals. It avoids to treat the imprinted resist as a sacrificial layer to transfer the pattern into the functional layer by etching in traditional semiconductor processes^27^,^28^,^29^. During the imprint processes, sequential imprinting and separation procedures are proposed to avoid high stress and minimize damages in the patterned structures^30^. By optimizing imprint parameters and synthesis conditions of the imprint resist, a series of high-quality photonic nano scale crystals are achieved without further annealing, post brake and other procedures assistances. The topography and robustness description indicates highly ordered hole and stripe groove arrays with sharp edges.

The polarization characteristic is an important property for the integrated optical devices. The polarization modulation effect of pure silica photonic structures has not yet been widely investigated. Here, we prove that the polarization characteristic can be well modulated by changing the incidence angle and structure periodicity of the pure silica photonic nanostructures. Ultimately this may provide a new way to obtain polarized laser light by using silica photonic structures as the cavity mirrors thereby introducing a simple solution to modulate light in nano devices.

## Silica sol – gel nanoimprint resist synthesis

**Figure 1:**
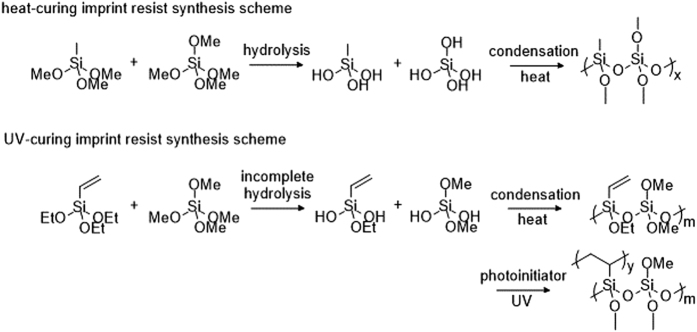
The synthesis process of the hot embossing resist and UV imprint resist.

As shown in Fig. 1, to obtain proper imprint sol-gel, methyltrimethoxysilanes (MTMS) and tetramethyl orthosilicate (TMOS) are mixed by using an appropriate MTMS:TMOS ratio followed by 0.3 mol of alcohol per mol of silicon in order to improve the miscibility of reactants and water. We conclude that the TMOS:MTMS ratio preferably is not less than 1:1 for high accuracy patterns. Accordingly, a 1 mol/L formic aqueous solution is injected as an acid catalyst with a water/alkyl group ratio of 1:1. Then the mixture is stirred at 36 °C for 120 min. After the reaction is finished, slight excess water is added into the mixture to reach 7 mol water per mol of silicon, ensuring the alkyl group’s complete hydrolysis. After that alcohol is added to dilute the mixture to a concentration of a not more than 1 mol Si/kg hydrolysis product. The imprint resist is obtained and should be stored below −25 °C for at least 24 hrs before use. During the nano imprint process solvent and water in the imprint resist are being removed by heating and spinning, promoting the reaction to go on the right way. To inhibit condensation reactions, the sol-gel is diluted with alcohols and stored at −25 °C, while the substitution reactions still do proceed. However, dilution decreases the viscosity of the imprint resist significantly, causing the thickness of the spin-coating film to drop and thereby partly affect the patterns precision. We demonstrate an optimal dilution mixture consisting of 1 mol Si/kg.

UV-curing imprint resist is fabricated on the basis of heat-curing the imprint resist with a fast curing rate. VTEO (triethoxyvinylsilane) is the most essential monomer substitution for MTMS to fabricate UV-curing resists. Under the action of ultraviolet photo initiators, carbon-carbon double bonds (-CH = CH2) on the VTEO monomers open extremely rapidly and the addition of polymerization finishes in a very short period of time. Similar to the synthesis process of heat-curing resist, a certain amount of VTEO and TMOS are mixed using an appropriate ratio. The degree of polymerization (DP) of polymerization products is determined by the proportion of carbon-carbon double bonds in the pre-polymer. However, the non-removable organic groups have a negative effect on optical and thermal properties after curing. The optimal ratio of TMOS to VTEO is 1:1. Accrodingly, 2 mol of water per mol of siloxane are added in a flask. The water was previously acidified with 1 mol/L formic acid. Unlike heat-curing the imprint resist, a small amount of water results in an incomplete hydrolysis of siloxane contributes to form linear oligomers with better mobility. Next, the mixture is stirred at 50 °C for 180 min and then let reacting at reduced pressure for 120 min. The imprint resist is fabricated from this mixture by adding a photo initiator (3 wt%). The final product is stored below −25 °C for the same reason as mentioned before for heat-curing the imprint resist.

## UV – Nanoimprint process

A UV silica resist layer is formed on a 4 inch diameter silicon wafer by one step spin coating, which ensures better homogeneity and less chance of “spin spokes”. The thickness of the nano imprint resist is about 500 nm. Optimum working parameters are summarized as follows: Firstly, set the spinning time as 10 sec, spinning speed as 500 rpm and spinning acceleration as 1000 rpm/sec to ensure resist uniformly distributed on the substrate. Especially, in the first step, an oscillation is necessary for rotation direction to avoid a thicker layer. Then the working time is set at 1 s, the spinning speed at 2000 rpm and the spinning acceleration at 2000 rpm/sec to reduce edge beads. The end step is to set the running time as 20 s, the spin speed at 300 rpm and the spin acceleration at 200 rpm/sec to reach the required thickness. Subsequently, soft working stamps with certain patterns are pulled to gradually contact the nano imprint resist from one side to another side followed by an ultraviolet curing and stripping process. The UV curing process has a great influence on the stripping process that determines success or failure of the whole imprint process. If the exposure time is too short, the imprint resist will not be completely cured, making it hard to get the high quality nanostructure. By contrast, if the exposure time is too long, the soft stamp and substrate will stick together and the stripping process cannot be completed. Sequential tests demonstrate that the optimal pre-UV contact time, post UV contact time and exposure time is 5 s, 10 s and 480 s under the power density of 41.5 mW/cm^2^. After all procedures are finished properly, the silica sub-micron nanostructures are transferred onto the silica substrates. It is important to note that the whole operation from the beginning of the imprint process to the end of the spin-coating needs to be completed in 30 s to prevent the resist curing.

## Results and Discussions

**Figure 2:**
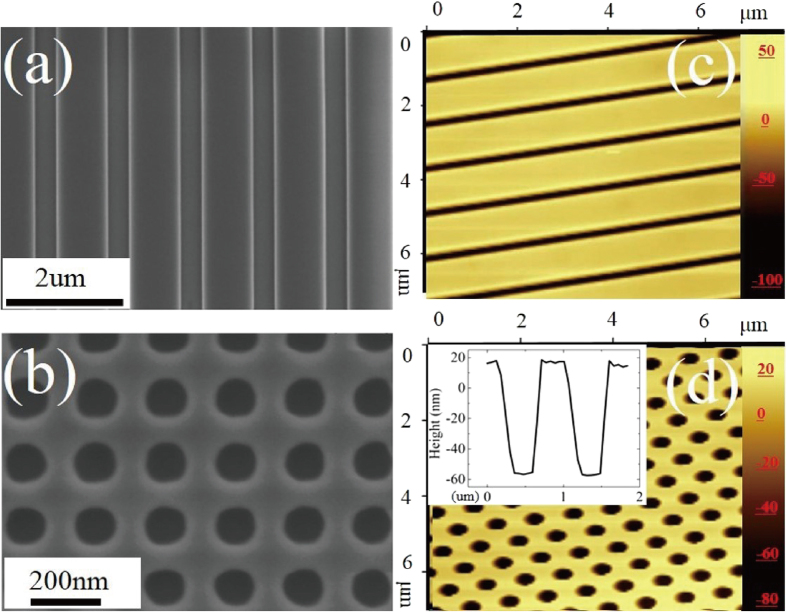
(**a**,**b**) SEM images of the photonic structures (**a**) the line width (LW) is 855 nm (**b**) the diameter (Dia) is 110 nm (**c**,**d)** AFM images of the photonic structures (**c**) depth is 74 nm, LW = 180 nm (**d**) depth is 75 nm, Dia = 430 nm.

The imprinted patterns present good periodicity, smooth surfaces and a uniform aspect ratio. As shown in Fig. 2(a,b), the line width of stripe nanostructures is 850 nm, the diameter of the hole nanostructures is 110 nm. The line width and diameter compared to the feature size of the original Si stamp is accurate within 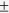
5 nm. The minimum line widths and diameters of the patterned structures is around 95 nm and 110 nm, respectively.

The aspect ratio and flatness is a key factor in evaluating the quality of photonic crystals and directly affects its further applications. The depth and surface roughness is checked by AFM (XploRA-Raman-AFM system, Horiba) as shown in Fig. 2(c,d). The AFM images show no obvious contrast difference in the whole area, which reveals a smooth surface. The depth is 74 nm, respectively 75 nm for stripe and tiny hole nanostructures. The line width and diameter is 320 nm, respectively 430 nm. The relative error for the depth and aspect ratio is around 4.1% and 6.2%, which is analyzed statistically by the corresponding AFM-generated profile, just like the insert shown in Fig. 2(d). The fluctuation of depth and aspect ratio is attributed to two aspects: first, the aspect ratio of the original mask has a slight fluctuation, which is mainly caused by the inhomogeneity of the etching process; second, to ensure a smooth test progress of the completed imprint pattern, the 4 inch silicon wafer with imprinted pattern is changed a little by the stress in the local area during cutting of the sample. The uniform aspect ratio and smooth surface can be attributed to the optimal nano imprint parameters mentioned above. In addition, the light dispersion experiment is also done to provide the validation as shown in Supplementary S1 and S2. The elements distribution and mapping by the Energy Dispersive Spectra (EDS) (Supplementary Figure S3 and Table S1) have proven that the main component is pure silica without another element after UV light exposure. The component distribution is uniform at different imprinted areas.

### Hot embossing imprint process

**Figure 3:**
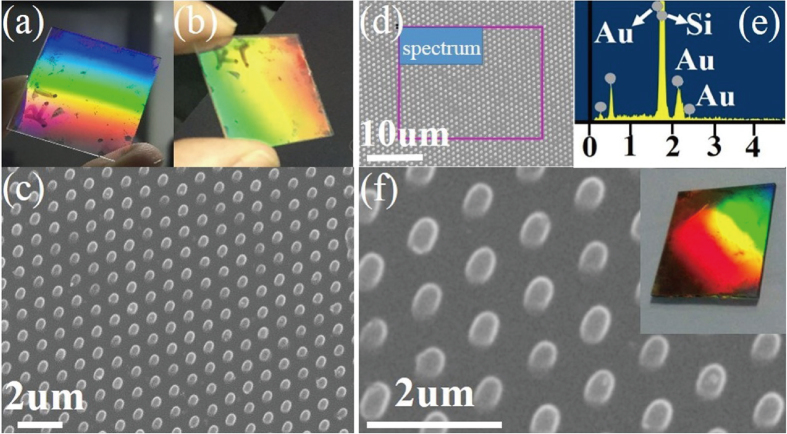
The real Optical image of the imprinted wafer (**a**) the transmission mode (**b**) the reflection mode (**c**) the SEM image of the cylinder nanostructures without gold nanoparticles on the surface (**d**) a low magnification SEM image of the cylinder nanostructures with a 5 nm gold film on the surface (**e**) the Energy Dispersive Spectroscopy corresponding to the area marked with the square in (**d**,**f**) high magnification SEM image, the insert is the real optical image of the imprinted wafer.

Silica sol-gel (0.3 ml) is spin-coated on glass substrate with dimensions of 2 cm*2 cm by spinning for 6 s at 500 rpm. Next, the stamp is put into the sol-gel by capillary force (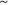
mT) by hand. After that, the film is cured at 65 °C for 2 min on a hot stage followed by imprint at a temperature as high as 100 °C. Finally, the stamp is released after the stage has cooled down to room temperature. The applied stress induced by hand can be well released, so there is no obvious defects and this nanostructure can be used to achieve good diffraction images in both transmission and reflection mode, which is shown in the real light patterns of Fig. 3(a,b). A SEM image of the nano cylinders shown is in Fig. 3(c). In order to enhance the reflection efficiency, a 5 nm gold nanoparticle coating was sputtered onto the surface of a cylinder pattern, the SEM image of which is shown in Fig. 3(d). The gold particle is randomly distributed on the surface of the structure and it does not affect the shape of the structure, which is proven by the high magnification SEM image shown in Fig. 3(f). The selected area EDS result shows a strong Au peak, as illustrated in Fig. 3(e). Theoretical results demonstrate that the reflection efficiency can be enhanced by 10–15% or more. Imprinted nanostructures on a larger glass substrate are shown in Supplementary Figure S4. In addition, the durability and damage threshold is also tested and the results shown in Supplementary Figure S5.

**Figure 4:**
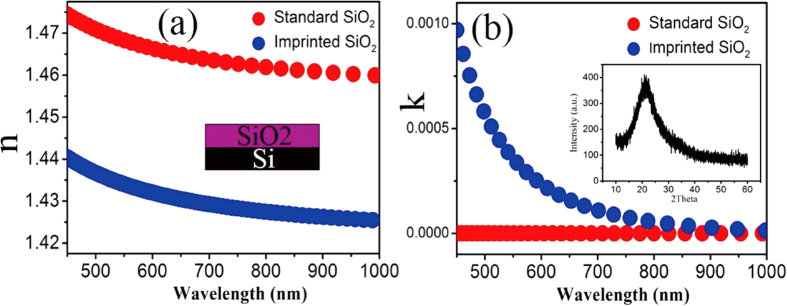
(**a**) Refractive index n at different incident wavelengths with an inset fitting model (**b**) extinction coefficient at different incident angles, with an XRD spectrum inset of the imprinted silica sample.

2-D silica photonic crystals can be used to manipulate light distribution and modulate light polarization. The refractive index (*n*) of the silica material is an important parameter for optical applications. For further clear description of the imprinted material, the optical factors *n* and *k* are acquired by ellipsometry (Auto Smart SE, Horiba). A comparison between the imprinted and the standard silica wafer is shown in Fig. 4. It can be seen that the refractive index *n* of the imprinted silica is in the range 1.44 to 1.42 with increasing wavelength from 450 to 1000 nm. It is around 1.43 at 632 nm. By comparison, the refractive index n of a standard silica wafer as obtained by magnetron sputtering changes from 1.47 to 1.45, as the red and blue lines show in Fig. 4(a). The inset of Fig. 4(a) is the fitting model of the matrix material SiO_2._ The imprinted silica shows absorption at the ultraviolet band, but the extinction coefficient is zero for standard silica. This is due to pre-polishing the standard silica wafer. In essence, the difference of the refractive index between imprinted and standard samples is mainly due to the difference in the structure and purity. XRD results in Fig. 4(b) show that the imprinted silica is amorphous. Furthermore, there is no big *n* and *k* difference at different locations, as shown in Supplementary Figure S6. The dielectric constant of imprinted silica is close to that of the silica dielectric layer in FETs, solar cells and other devices as well as manufacturing processes^31^,^32^,^33^. Accordingly, imprinted silica has appropriate insulation properties.

**Figure 5:**
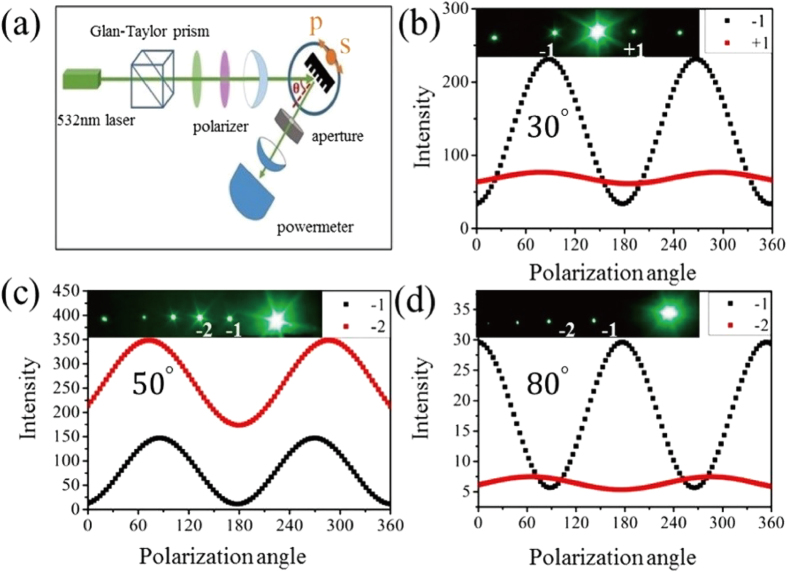
(**a**) Diagram of the angle-dependent-polarization measurement setup (**b**) the power variation of −1 and +1 diffraction order with the change of polarization angles, the incident angle is 30° (**c**,**d**) power variation of −1 and −2 diffraction order with change of polarization angles, the incident angles are 50° and 80°. The line width of the structure is 570 nm.

In the end, the polarization characteristics of the reflected light can be modulated by changing the incident angle. The polarization testing system as shown in Fig. 5(a), uses 532 nm laser light emitted from a solid–state laser passing through a Glan-Taylor prism and a quarter-wave plate (532 nm), which produces circularly polarized light, which is focused on the nanostructure by long focal-length lenses. A polarizer is used to change the polarization direction and a power meter (Gentec – EO, PH100-Si-HA-D0) is used to check the intensity of the reflected light. This intensity changes with the polarization angle and is shown in Fig. 5(b–d). The period and line width of the 2-D silica photonic crystal is 2 um and 570 nm. Also, real diffraction patterns at different incidence angles are acquired by a high-resolution CCD camera as shown in the inset of Fig. 5. For the incident angle of 30°, the reflection intensity of the *p*- wave is greater than that of the *s*–wave. The degree of polarization, DOP = (Pmax − Pmin)/(Pmax + Pmin), is 0.07, 0.73 and 0.12 for 0, −1 and +1 order, respectively. However, when the incident angle becomes 80°, the polarization direction of the first diffraction order changes by 90°. The *s*–wave intensity becomes a maximum and the *p*–wave intensity decreases to a minimum. The DOP is 0.73 and 0.22 for −1 and −2 order. The numerical simulations are performed by Rsoft 8.1 based on Rigorous Coupled Wave Analysis (RCWA) as shown in S6. The change trends are consistent with the experimental results, but the DOP of different diffraction order shows a little difference between the experimental and the simulation results. Notably, an unusual diffraction pattern is found at 50°, as shown in Fig. 5(d). The energy of the −2 diffraction order is greater than that of −1 order. The detected power is 33.2 μW and 71 μW when the polarization is perpendicular to the structure. The reason of the DOP mismatch and the unusual diffraction phenomenon can be attributed to high coherence laser light, which is sensitive to the slope caused by the thickness fluctuation of the silicon wafer. More quantitative explanations are shown in the Supplementary Figures S7, S8 and S9 and Table S2. However, this uncommon situation is not a universal phenomenon for the whole photonic nanostructure. Absolutely, the silica photonic crystal nanostructure not only can modulate the polarization properties at the visible band but also at the IR band. Therefore, the 2-D silica photonic nanostructure can be used as a functional layer to modulate the polarization characteristics. In addition, it can be regarded as the traditional dielectric layer used in semiconductor devices. All in all, a imprinted 2-D silica photonic crystal can promote the development of optoelectronic devices and may lead to more brand-new and extensive applications, such as quantum devices.

## Conclusions

In summary, a detailed introduction to synthesized silica sol – gel nano imprint resists and important imprint parameters for UV and hot embossing nano imprint processes are highlighted. Large area 2-D silica photonic crystal nanostructures with excellent periodicity and a consistent aspect ratio can be fabricated within five minutes, thereby improving efficiency in many optical fields such as an optical grating, light-wave and optical circuits. Furthermore, glass substrates with imprinted cylindrical structures show good transmission and reflection characteristics. In addition, the refraction index of imprinted silica is close to that of dielectric layers used in nano devices and the polarization characteristics can be modulated by stripe photonic nanostructures through changing the incident angle. So, 2-D silica nanostructures not only can be used as dielectric layers but also as a functional layers to improve the performance of the devices. Besides, the silica photonic may have huge potential applications in nano - laser, 3D display and novel LED technology.

## Method

The focus of this paper is the fabrication method of two dimensional silica photonic crystals based on UV nano imprint lithography and hot imprint. The specific methods are described in the body of the paper

## Additional Information

**How to cite this article**: Guo, S. *et al*. The polarization modulation and fabrication method of two dimensional silica photonic crystals based on UV nanoimprint lithography and hot imprint. *Sci. Rep*. **6**, 34495; doi: 10.1038/srep34495 (2016).

## Supplementary Material

Supplementary Information
