# Supplementary material for: The polarization modulation and fabrication method of two dimensional silica photonic crystals based on UV nanoimprint lithography and hot imprint
Source: Sci Rep. 2016 Oct 4;6:34495. doi: 10.1038/srep34495 (PMC5048289; doi:10.1038/srep34495)
Supplement: Supplementary Information [file srep34495-s1.doc]

***The polarization modulation and fabrication method of two dimensional silica photonic crystal based on UV nanoimprint lithography and hot imprint***

***Shuai Guo^1^, Liang Liang^1^, Chunhui Niu^2^, Ke Chai^1^, Yaqing jia,^3^ Fangyin Zhao^1^, Ya Li^1^, Bingsuo Zou^1^ and Ruibin Liu^1*^***

*^1^Beijing Key Laboratory of Nanophotonics and Ultrafine Optoelectronic Systems, Institute of Physics, Beijing Institute of Technology, Beijing 100081, China*

*2 School of Instrument Science and Opto-electronic Engineering, Beijing University of Information Science & Technology, Beijing 100192, China*

*3 Beijing institute of metrology, Beijing 100029, China*

**Corresponding Author Tel.: +86 10 68918188.*

*E-mail Address:* [*liuruibin8@gmail.com*](mailto:liuruibin8@gmail.com)

***Surpporting Information***

Light dispersion effects can reflect the quality of silica photonic crystal~~s~~ structures. The imprinted wafer is placed under a dark-field microscope (Olympus BX51M). Different objective lens means different amplification and N.A, as the simple schematic diagram shown in figure S1 (a) indicates. The periodic silica nanostructures with different line widths present specific colors. The real CCD images and the corresponding high magnification CCD images are shown in figure S1 (b) - (e) (the magnification of the objective lens is100×, NA=0.92). As the low magnification (the magnification of the objective lens is 5×, NA=0.10) CCD images (the insert in Figure) show~~s~~, the stripe nanostructures with line width 469 nm, 357 nm and 569 nm present emerald green, yellow and purple color. The tiny hole nanostructures are presented by blue color (the magnification of the objective lens is 20×, NA=0.40).


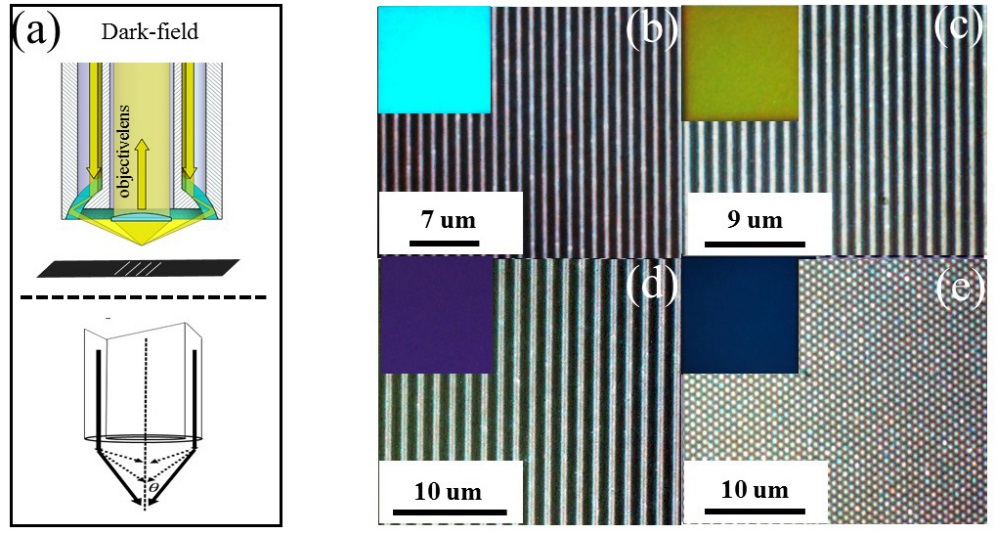


Figure S1 (a) Diagrams of dark-field microscopy (b-e) the high and low magnification images of different silica photonic crystal nanostructures

Furthermore, the quantitative diffraction characteristic is also investigated. The white light irradiates the photonic crystal structures at a certain angle (30°-50°) and a scanning detection system is utilized to collect the diffracted light. The diagram of the system is shown in figure S2 (a), in which the structure width is 570 nm. It is placed on a two-dimensional stage and the light emitted from the Xenon lamp is focused on the microstructure at a certain angle to ensure the focus spot can cover the imprinted nanostructure. On the other side, the optical image is firstly recorded, as shown in figure S2 (b) (the distance between the optical screen and the light source is about 6 cm). Then a fiber optic spectrometer (AvaSpec-ULS 2048) is utilized to analyze the spectrum information (the distance between the fiber and the microstructure is around 1 cm). The normalized low- and high-resolution spectra are shown in figure S2 (c), (d).


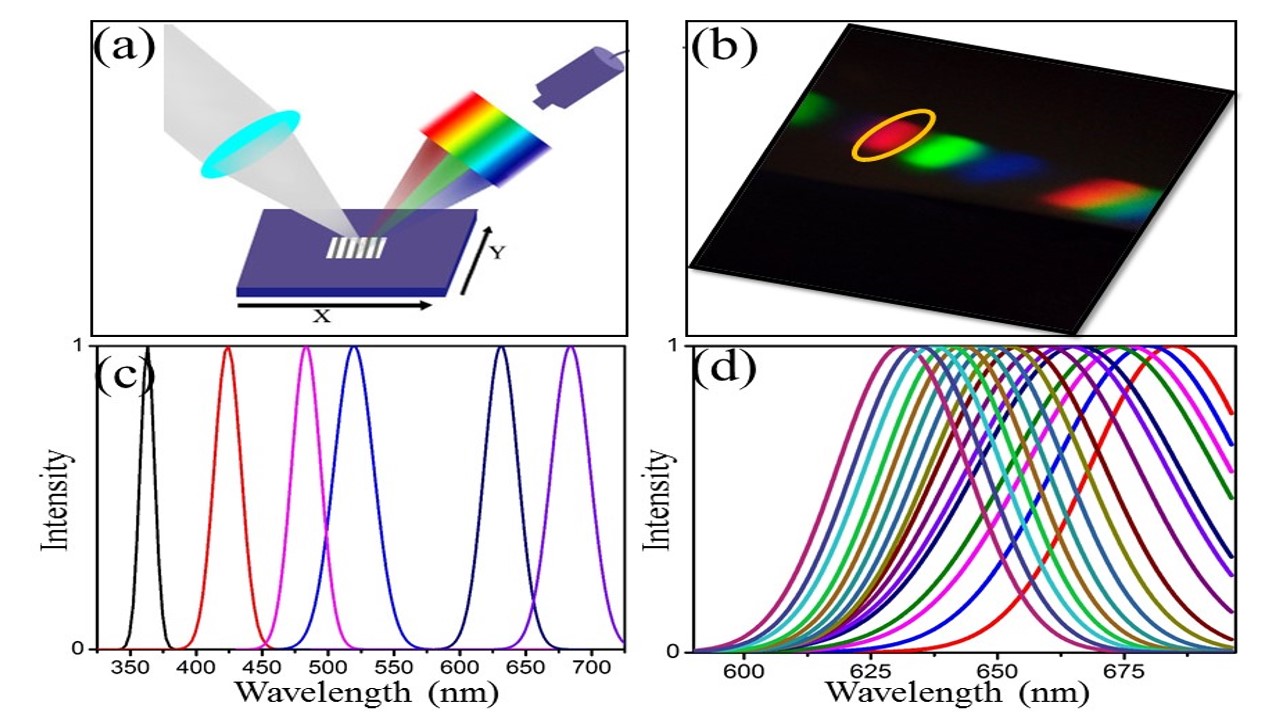


Figure S2 (a) Diagram of the testing system (b) the real diffraction image (c) (d) the low-resolution and high-resolution spectra.


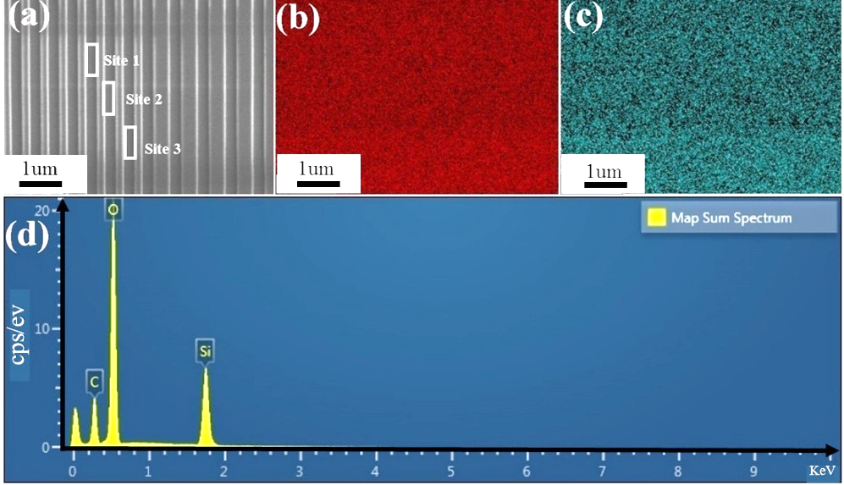


Figure S3 (a) SEM image of the stripe structure with different line widths (b) (c) the mapping image of the oxygen and silicon element (d) the sum energy dispersive spectrum

**Table S**1 element composition of the nanostructures

| **Element** | **Line Type** | **Apparent Concentration** | **k Ratio** | **Wt %** | **Wt % Sigma** | **Standard Label** |
| --- | --- | --- | --- | --- | --- | --- |
| **C** | K series | 0.93 | 0.00931 | 14.40 | 0.16 | C Vit |
| **O** | K series | 13.88 | 0.04670 | 49.60 | 0.18 | SiO2 |
| **Si** | K series | 4.64 | 0.03676 | 36.00 | 0.19 | SiO2 |
| **Total:** |  |  |  | 100.00 |  |  |

Figure S3 and table S1 demonstrate that the component of the imprinted nanostructures is silica and there is no other elements.


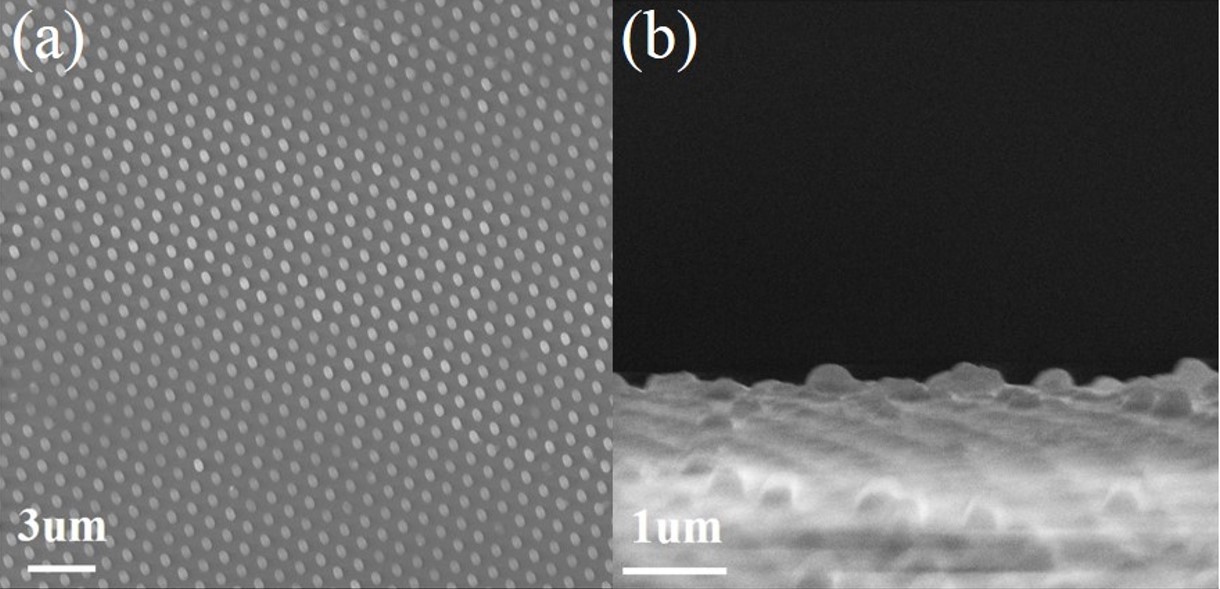

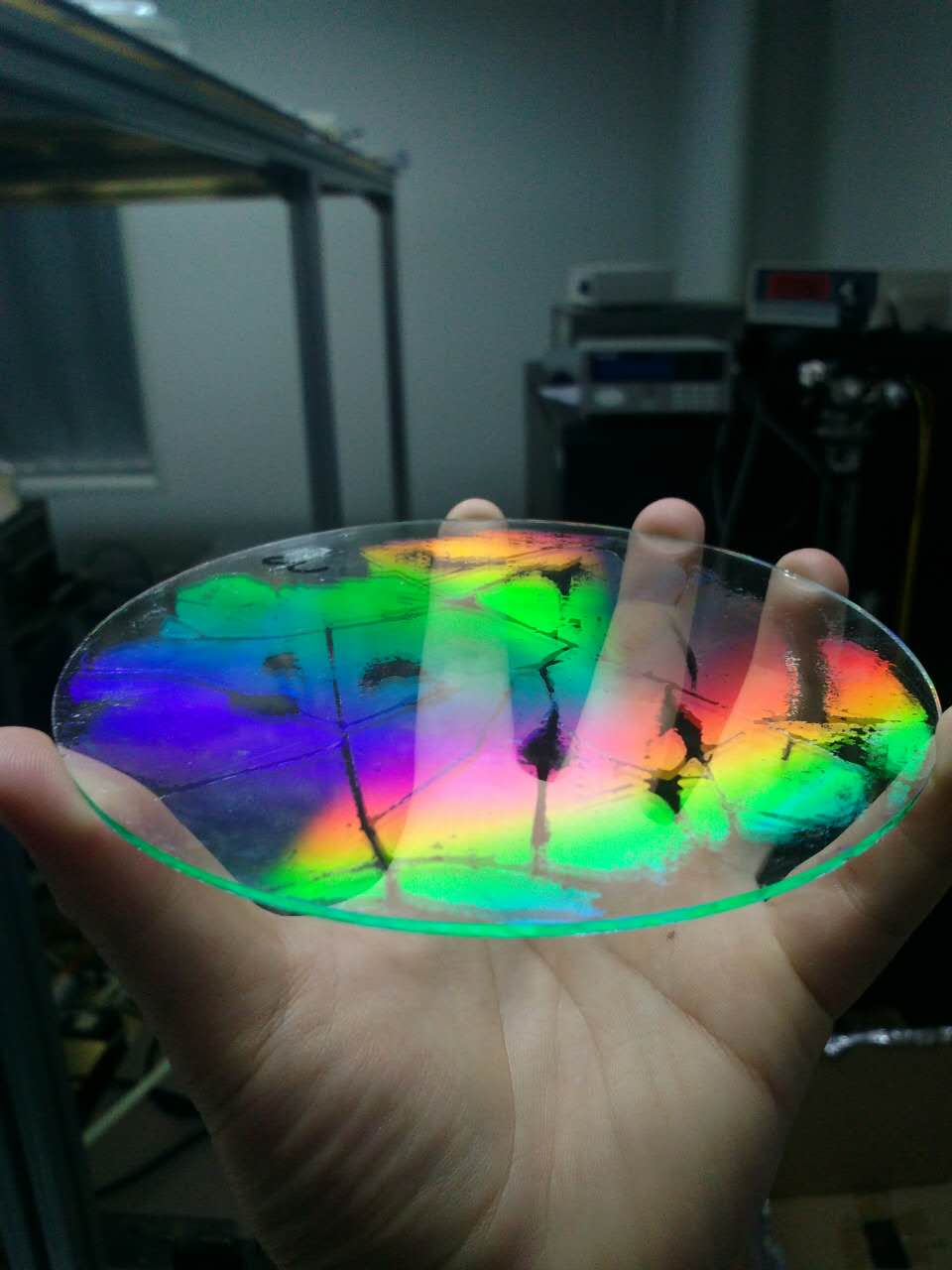


Figure S4 (a) SEM image of a hot embossing imprint pattern (b) the diffraction pattern on a 4 inch diamter SiO_2_ substrate under fluorescent light in our lab (the dark line in the color strip is due to the periodic nanostructures, scratched artificially for comparison)

Figure S4 (a) is the SEM image of a hot embossing imprint pattern the structure of which has good periodicity. Figure S4 (b) is the diffraction pattern is a 4-inch SiO_2_ substrate under fluorescent light in our lab. The large 4-inch patterned chip could form the color strip, whatever works in transmission or reflection mode.


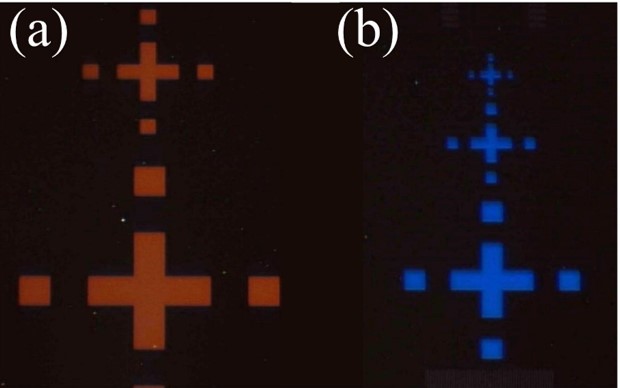


Figure S5 (a),(b) Dark field CCD image at different magnification

After one year in the atmosphere, the nanostructures still show uniform color under a dark field optical microscopy and the damage threshold also be checked by using 532nm pulsed laser (Nd:YAG 532 nm, Newwave Inc., US ), the threshold is about 15 MW/cm^2^


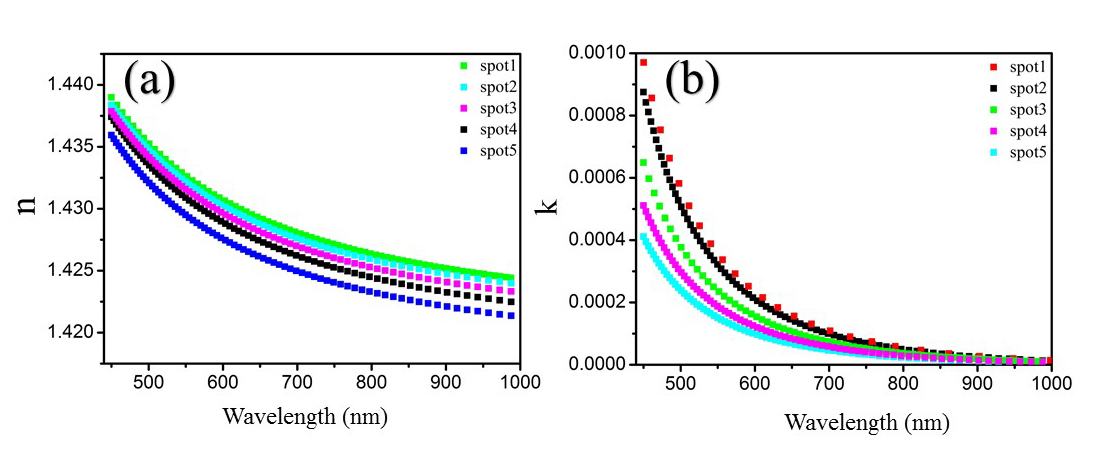


Figure S6 (a), (b) Refractive index and extinction coefficient at different positions

In order to verify the difference of optical factors, the refractive index and extinction coefficient at five random spots has been checked. The maximum and minimum value at 450 nm is 1.4389 and 1.4359. The relative error is 0.1% for the refractive index. The results prove that there are no big differences in the whole imprinted area.


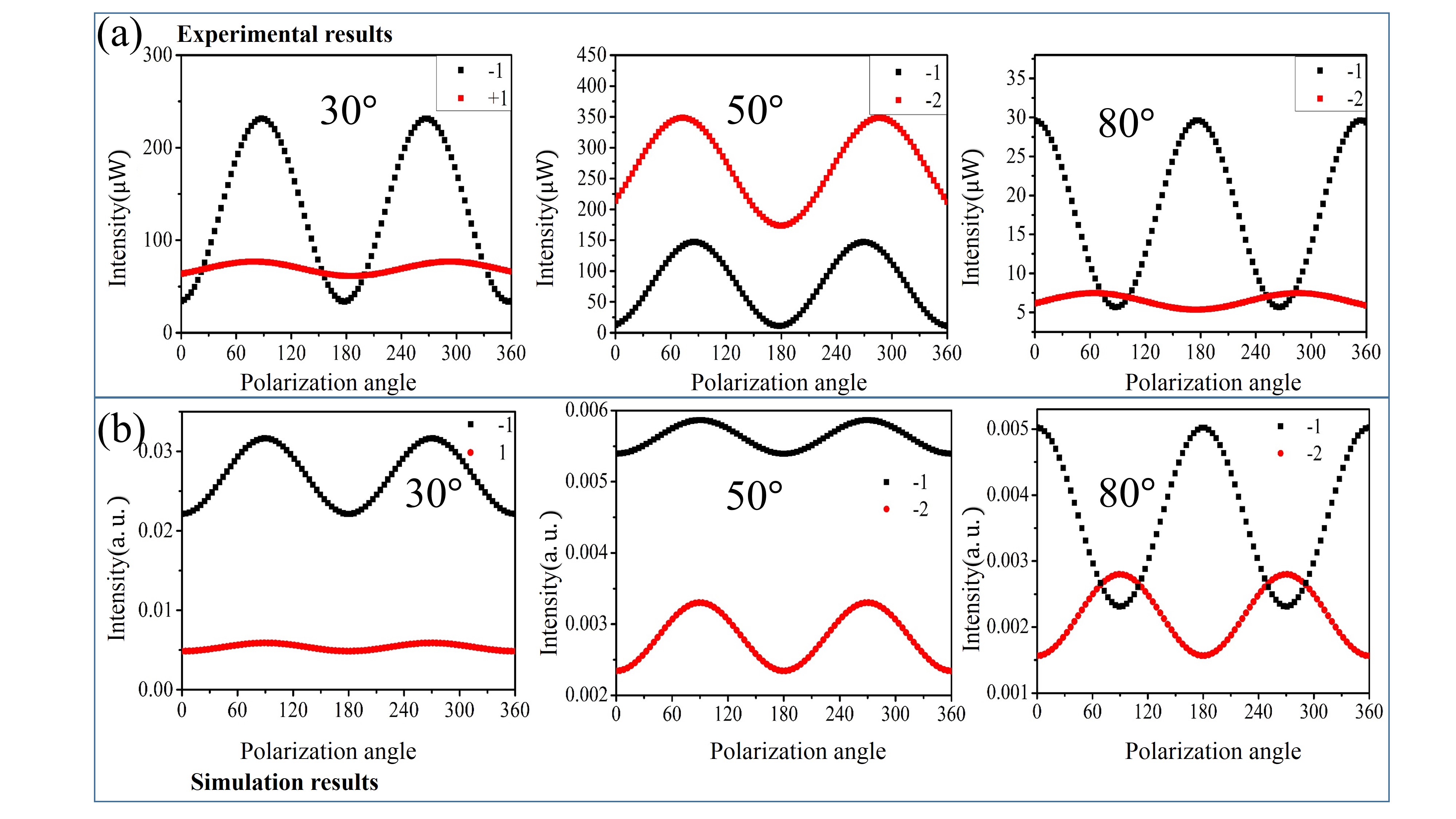


Figure S7 The power variation of different diffraction orders as function of the polarization angle at different incident angles 30^o^, 50^o^ and 80^o^ (a) the experimental results (b) the simulation results.

Rsoft 8.1 based on Rigorous Coupled Wave Analysis (RCWA) is used to simulate the polarization modulation effects the results of which are shown in figure S6 (a), (b). The simulation results are consistent with the experimental results, showing the same trend. But there is a mismatch of the degree of polarization, which is explained in the following.


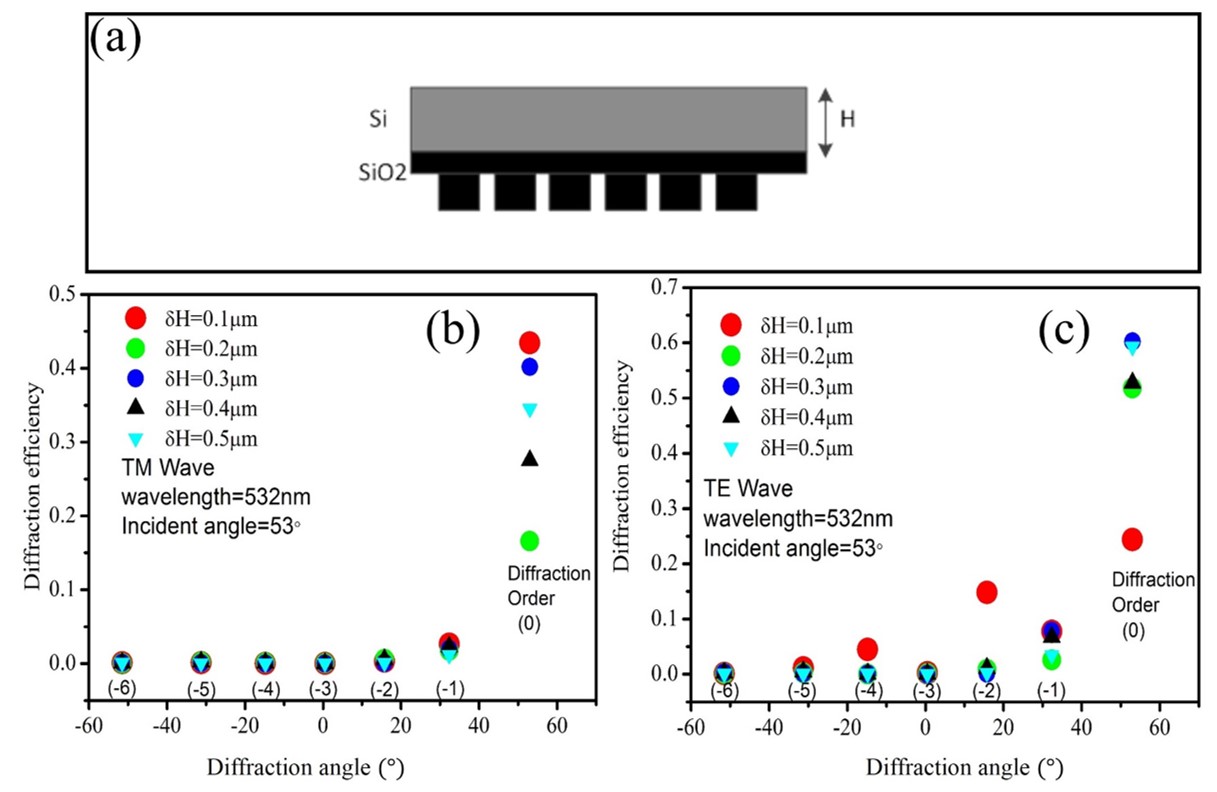


Figure S8 (a) Diagram of the imprinted pattern on a flat silicon wafer (b), (c) the diffraction efficiency at different diffraction order with changes of the wafer thickness (δH).

The schematic diagram of the normal structure is shown in figure S7 (a). With the variations of the wafer thickness, the diffraction efficiency with no apparent changes is demonstrated. The corresponding results of zero diffraction order with the highest diffraction efficiency for TE and TM waves are shown in figures S7 (b) and (c).


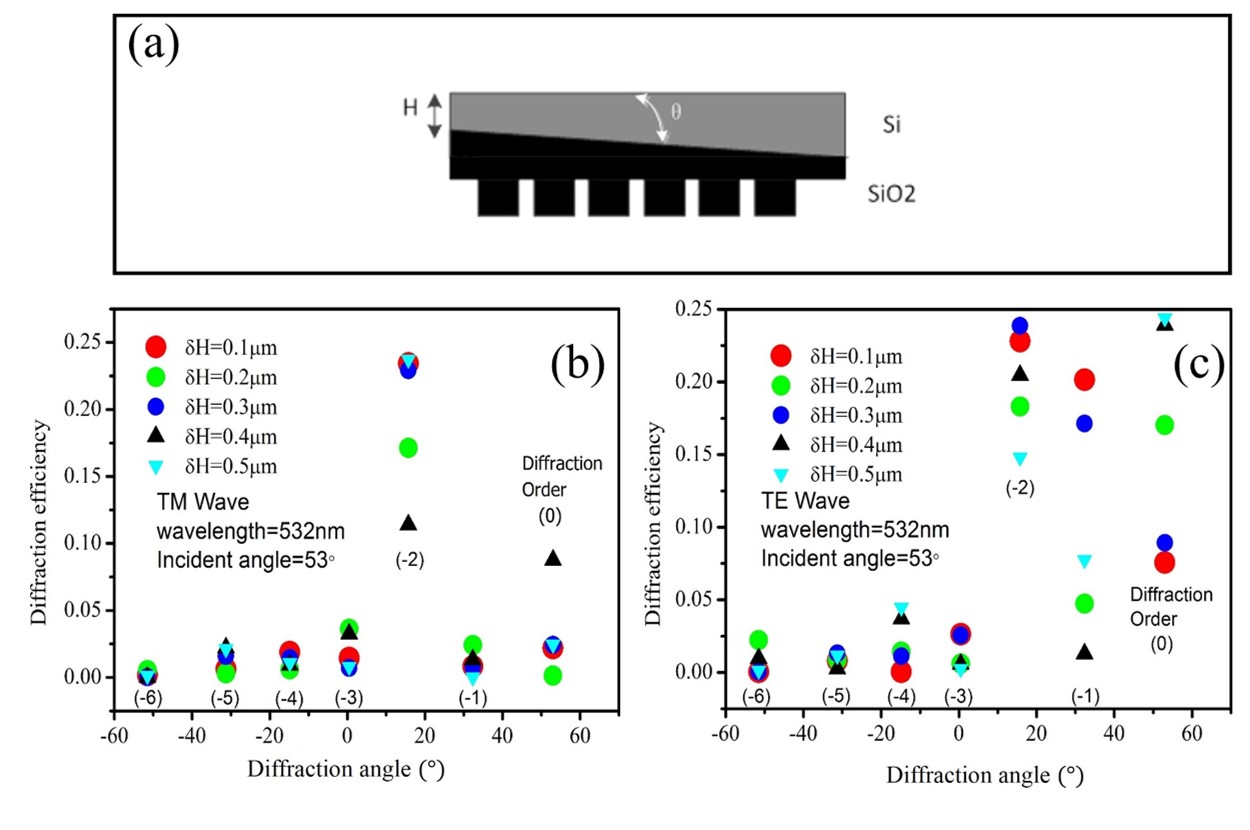


Figure S9 (a) Diagram of the imprinted pattern on a gentle dip wafer, θ = 5° (b) and (c) the diffraction efficiency of different diffractions with the change of short edge thickness (δH).

Compared to the normal situation, Figure S8 (a) shows the imprinted pattern on a gentle dip silicon wafer. Due to the presence of inclination, the relative intensity of the different diffraction order shows an obvious change of the TE and TM wave. Figures S8 (b) and (c) show either -2 or 0 diffraction orders with high efficiency. There are no obvious differences. In view of the light source (high coherent 532nm laser), this diffraction effects become more sensitive to the variations in thickness, so that the diffraction efficiency shows ~~a~~ big changes even at small thickness variations.

**Table S2 the degree of polarization of different wafer thicknesses**

| **DOP** | **δH=****0.1μm** | **0.2μm** | **0.3μm** | **0.4μm** | **0.5μm** |
| --- | --- | --- | --- | --- | --- |
| **-6** | 0.649986 | 0.607317 | 0.173477 | 0.988874 | 0.768809 |
| **-5** | 0.130433 | 0.476285 | 0.094348 | 0.797594 | 0.790547 |
| **-4** | 0.948549 | 0.401941 | 0.1328 | 0.598815 | 0.545205 |
| **-3** | 0.295093 | 0.70979 | 0.564525 | 0.696512 | 0.150361 |
| **-2** | 0.01338 | 0.032973 | 0.020097 | 0.284462 | 0.073858 |
| **-1** | 0.92198 | 0.326597 | 0.913741 | 0.015671 | 0.995625 |
| **0** | 0.549185 | 0.984424 | 0.572488 | 0.463796 | 0.81365 |

The simulation results in table S2 show that the thickness differences induce big variation in the degree of polarization (DOP) for different diffraction orders. Similarly, there is no obvious rule to follow.
